# Breast abscess due to *Salmonella* Typhimurium in a patient with rheumatoid arthritis: a case report

**DOI:** 10.1186/s12879-016-1659-z

**Published:** 2016-07-22

**Authors:** Irmak Baran, Neriman Aksu, Altan Aksoy

**Affiliations:** Medical Microbiology Department, Ankara Numune Training and Research Hospital, Hacettepe Mahallesi Talatpasa Bulvari No:44, Altindag, 06100 Ankara Turkey

**Keywords:** *Salmonella* Typhimurium, Breast abscess, Rheumatoid arthritis, Extraintestinal salmonellosis, Case report

## Abstract

**Background:**

This is the first report of breast abscess due to *Salmonella enterica* serotype Typhimurium. *Staphylococcus aureus* is known as the most common cause of breast abscess. *Salmonella* spp. may occasionally form localized abscesses after dissemination to various organ systems following a bacteraemia. But breast abscess related to *Salmonella* spp is a very rare complication.

**Case presentation:**

A 43-year-old female patient referred to our hospital with a lump, fever and mild pain in her breast. The patient was not pregnant or lactating at that time. She had a history of rheumatoid arthritis for 5 years and was under immunosuppressive therapy. Ultrasonography of the breast revealed an abscess. The abscess was drained and sent for culture to medical microbiology laboratory. The microorganism was identified as *Salmonella enterica* serotype Typhimurium and found to be sensitive to all antibiotics tested. The patient was cured after surgical debridement and antibiotic therapy. The abscess did not recur again.

**Conclusions:**

This case is presented to draw attention to non-typhoidal *Salmonella* as rare causes of breast abscess and submission of specimens to the microbiology laboratory for accurate diagnosis and treatment especially in patients with underlying immunosuppressive diseases.

## Background

Infections caused by bacteria belonging to *Salmonella* genus can be presented in five different clinical forms. These are enteric fever, sepsis, enterocolitis, localized organ disease and chronic carrier state [1, 2]. *Salmonella* spp. are facultative intracellular bacteria. But they can also live freely [3]. They can survive in reticuloendothelial system macrophages and can spread by circulation. If the infection is untreated or resistant to treatment, circulating bacteria can settle in various organs and cause extraintestinal disease [2, 4, 5].

The pathogenesis of extraintestinal *Salmonella* infections is affected by inoculum size of the ingested bacterium, virulence of the strain, local defence mechanisms and host’s immune response [6]. Advanced age, gastric surgery, immunosuppressive diseases (HIV infection, etc.), immunosuppressive therapy malignancy (metastatic cancer, lymphoma, etc.), hemoglobinopathies, iv drug use, diabetes and passed trauma at the site of infection are risk factors blamed for localized extraintestinal *Salmonella* infections [2, 3, 7–9].

*Staphylococcus aureus* is the most common cause of breast abscess. Furthermore, streptococci, gram-negative bacilli, and anaerobes may cause breast abscess [1, 7, 10, 11]. Breast abscess due to *Salmonella* is rare and late complication of enteric fever [4]. Breast abscess were reported to develop after *Salmonella enterica* serotype Typhi and Paratyphi infections. But breast abscesses related to the non-typhoidal *Salmonella* are extremely rare [1, 8].

Here we present a case of breast abscess due to *S*. Typhimurium in a non-lactating 43-year-old female patient with rheumatoid arthritis (RA) and we have made a brief review *Salmonella* breast abscess cases in literature so far.

## Case presentation

A 43-year-old female patient referred to our hospital’s emergency department in August 2014 with complaints of fever and severe pain in right breast. She noticed a soft lump and mild pain in her breast a week back. The intensity of pain increased gradually and for the last 2 days and her temperature increased. The patient had a history of 5 years of RA. The patient was not pregnant or lactating. She had three children with the last one delivered 6 years back.

On physical examination, the right breast was tender and swollen. A mobile, sensitive, soft, fluctuating mass of around 4 by 5 cm located in the right lower quadrant was palpable. The mass was not fixed to the upper skin. The upper skin was warm and erythematous. There was no nipple retraction or discharge from the nipple. Axillary lymph nodes were not palpable. The patient’s respiratory, digestive, nervous system physical examinations were normal. Physical examination of the musculoskeletal system revealed swelling in 2^nd^ and 3^rd^ proximal interphalangeal joints of the right hand and swelling and limitation of motion in the right knee.

Ultrasonography (USG) of the right breast revealed a heterogeneously hypoechoic deep-seated irregular collection of approximately 40×38 mm size. The patient was diagnosed with a breast abscess. The abscess was drained by USG guided drainage, and oral amoxicillin clavulanic acid 625 mg bid therapy was started empirically.

The drained fluid was sent to medical microbiology laboratory in a tightly capped, sterile container. On macroscopic examination, it was observed that the material had yellowish cream colour and dense consistency. Gram staining of the material showed abundant polymorphonuclear leukocytes and gram-negative bacilli. The material was inoculated on 5 % sheep blood and eosin methylene blue (EMB) agars plates. One of the sheep blood agar plates was incubated under anaerobic conditions. After incubation at 37 ° C for 24 h smooth, straight-edged colonies without hemolysis grew on sheep blood agar. On EMB agar, lactose-negative colonies were detected which tested negative for oxidase production. These colonies were identified as *Salmonella species* by Phoenix BD (Becton, Dickinson, USA) automated system and *Salmonella* group by Maldi-TOF MS (bioMérieux, France). The microorganism was identified as *Salmonella enterica* serotype Typhimurium according to the Kauffmann-White scheme by using specific antisera (Difco, Becton, Dickinson, USA). No anaerobic bacteria were isolated. The microorganism was found susceptible to ampicillin, ciprofloxacin, trimethoprim-sulfamethoxazole, erythromycin, chloramphenicol, ceftazidime, and ceftriaxone by antibiotic susceptibility testing with Phoenix BD (Becton, Dickinson, USA) automated system.

**Fig. 1:**
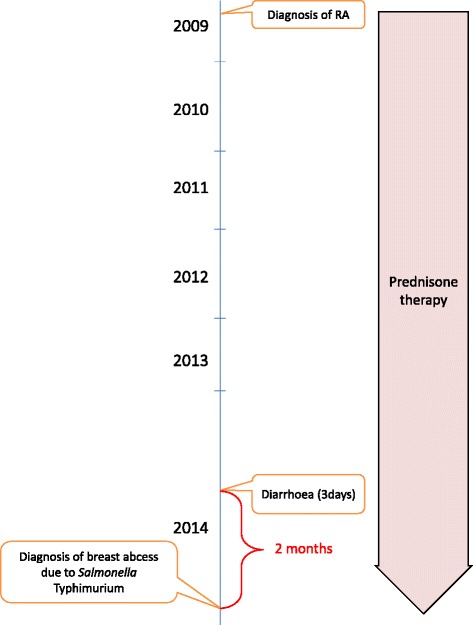
Timeline of events since the patient was diagnosed with Rheumatoid arthritis (RA)

The patient was called back to the surgical outpatient clinic to obtain a detailed history. It was learned that 2 months ago she had diarrhoea for approximately 3 days and did not receive any antibiotic treatment. The patient had RA for 5 years and was on prednisone therapy. There was no history of contact with animals or trauma to breasts. There was no history of similar swelling in the other breast; no history of diabetes mellitus, hypertension, bronchial asthma, and tuberculosis. When the patient’s laboratory tests were examined leukocyte count was 12,400/mm3 (83.9 % neutrophils), hemoglobin was 13.3 g/dL, fasting blood glucose was 108 mg/dL, erythrocyte sedimentation rate was 31 mm/h, CRP was 32 mg/l, RF was 28 IU/ml and anti CCP was 267.9 U/mL. Alkaline phosphatase, amylase, aspartate aminotransferase, alanine aminotransferase, gamma-glutamyl transpeptidase, bilirubin levels were within normal limits. HBsAg, anti-HCV and anti-HIV (ETI-MAX 3000 analyzer; DiaSorin S.p.A., UK), anti-HBc (Cobas 6000 analyzer, Roche Diagnostics, USA) tests were found to be negative. Chest X-ray was normal. Stool, urine, and blood samples were obtained for culture and found negative for *Salmonella* spp. Gruber Widal test was also found negative. Figure 1 shows a timeline of events.

The patient was fully treated with the surgical draining of the breast abscess under general anaesthesia and oral ciprofloxacin 500 mg twice daily. At the end of 2 weeks, the patient was fully healed with no clinical signs of abscess. The control examination with USG after 4 months showed no sign of recurrence of the abscess.

## Discussion

Pregnancy, lactation, breast malignancy, hematoma, advanced age, gastric surgery, previous local trauma and drug abuse are known predisposing factors for breast abscess [1, 2, 4, 7, 8]. The case we reported here did not have any of these risk factors. But our patient had RA as an underlying disease and was using immunosuppressive drugs. RA patients have an increased risk of developing infectious diseases. The increased risk of infection in RA patients found to be associated with three factors: The nature of pathophysiology of the disease, comorbid chronic diseases and usage of immunosuppressive therapy for the treatment of the disease [12]. In recent studies, it is observed that immune system (especially T cells) ages prematurely in RA patients and they become inadequate in defence against infectious agents [13–16].

The patient had no history of contact with animals. When the patient’s history was reviewed, it was revealed that the patient had an episode of gastroenteritis. We suppose the patient might have had a gastroenteritis related to *Salmonella*, but we cannot be sure since no stool sample was taken at the time of gastroenteritis. Focal salmonellosis is thought to occur secondary to bacteraemia after gastroenteritis. Patients with severe underlying conditions are known to be prone to focal infections [9]. The patient’s breast might have became infected during a transient bacteriemic episode originating from patient’s bowel and immunosuppressive state of the patient might have facilitated formation of the abscess. Localized *Salmonella* infection may occur after seeding of bacteria due to overt or silent bacteremia [1]. Secondary bacteremia develops in 3–8 % cases of non-typhoidal *Salmonella* infection, and localized infection may only occur 5–10 % of these [7]. *Salmonella* bacteremia in patients with severe immune system impairment found to be associated with increased risk of extraintestinal disease [7]. Previously extraintestinal *Salmonella* infections have been reported in patients who have had rheumatologic diseases (systemic sclerosis, systemic lupus erythematosus) and been receiving immunosuppressive therapy [2]. In our case, we think that having 5 years of RA and using immunosuppressive therapy might be contributing factors for formation of *S*. Typhimurium breast abscess.

*S*. Typhimurium had been isolated previously from septic arthritis, meningitis, hemorrhagic pleural effusion, brain abscess, parapharyngeal abscess [2, 9]. We have done a literature review, and we concluded that this is the first case report of a breast abscess associated with *S*. Typhimurium.

When we made a literature review we found that the reported cases of breast abscess are mostly due to *S.* Typhi and Paratyphi infections. On the other hand, breast abscesses related to the non-typhoidal *Salmonella* isolates are very rare [8]. Table 1 shows the reports of breast abscess cases due to *S.* Typhi and Paratyphi infections when a literature search using Pubmed and Medline was done. It is interesting that most of the cases were from India where these infections are endemic. Typhoid breast abscesses have been reported to develop in 0.9 % of the patients with generalized *S.* Typhi infections [1, 7]. The first reported case of breast abscess was by Thayer and Hazen, who isolated *S.* Typhi from the breast abscess of a young housemaid presenting to the Johns Hopkins Hospital, Baltimore in 1907 [17]. Madelung had stated that until 1923 there were only 30 cases of *Salmonella* breast abscess case reports in the literature. Madelung ve Erbslöh said that in the breast abscess cases overlying skin was not erythematous and *S.* Typhi isolated from abscess was not in pure colony form, it was often part of a mixed growth. But later reports stated the opposite [11, 18]. In our case, *S.* Typhimurium was isolated in pure culture form, and the overlying breast skin was erythematous. Klose ve Sebening stated in 1930 that mastitis develops in only 0.3 % of the patients with typhoid fever. But Pezinski examined 1196 cases of typhoid fever in 1937 and determined that frequency of breast abscess in the general population was 0.5 % and when only female patients were considered this frequency was 0.9 % [11, 18, 19]. Fernando et al. said that between 1970 and 2012, fewer than 15 breast abscess cases due to *S.* Typhi have been reported [4].

**Table 1:** Clinical features of reported cases of breast abscess due *Salmonella enterica* serotype Typhi and Paratyphi. (Source: PubMed, Medline)

Year of publication	Reference number	Author	Patient age	Gender	Underlying condition	Species	Unilateral/bilateral breast abcess
1907	[17]	Thayer and Hazen	16	Female	None	*S.* Typhi	Unilateral
1972	[18]	Barrett and MacDermot	43	Female	None	*S*. Typhi	Unilateral
1994	[20]	Lalitha and John	Unknown	Unknown	Unknown	*S*. Typhi	Unknown
2003	[5]	Jayakumar et al.	40	Female	Fibroadenoma of the breast	*S*. Typhi	Unilateral
2007	[21]	Delori et al.	54	Female	None	*S*. Typhi	Unilateral
2007	[22]	Mahajan et al.	Unknown	Female	Immunocompromised	*S*. Typhi	Unilateral
2009	[10]	Singh et al.	35	Female	None	*S*. Typhi	Bilateral
2011	[19]	Singh et al.	29	Female	None	*S*. Typhi	Bilateral
2011	[23]	Vattipally et al.	28	Female	None	*S*. Typhi	Unilateral
2012	[4]	Fernando et al.	33	Female	None	*S*. Paratyphi A	Unilateral
2012	[24]	Kumar et al.	60	Female	Diabetes Mellitus	*S*. Typhi	Unilateral
2012	[25]	Siddesh and Sumana	33	Female	None	*S*. Paratyphi A	Unilateral
2013	[11]	Banu et al.	40	Female	None	*S*. Typhi	Unilateral
2014	[26]	Ghadage et al.	31	Female	None	*S.* Paratyphi A	Unilateral
2015	[6]	Sood	37	Female	Diabetes Mellitus Type 2, hypothyroidism	*S.* Paratyphi A	Unilateral
2016	[27]	Elumalai and Seetharaman	Unknown	Unknown	Unknown	*S.* Typhi	Unknown

Breast abscess due to *Salmonella* spp. has also been observed in the neonatal period. Multi-drug resistant *Salmonella* isolation from breast abscesses has been reported. In a study Singh et al. retrospectively evaluated the reported cases of *Salmonella* breast abscess in the literature and concluded that most of the patients were between 23 and 45 years of age, immuno competent, non-lactating women. But they could not find a common predisposing factor [19].

There are few cases of breast abscess related to the non-typhoidal *Salmonella* isolates reported in the literature (Table 2). Edelstein et al. stated that until 1993 there were only six breast infection involvement cases related to non-typhoidal *Salmonella* isolates reported in the literature. Only two cases of these were breast abscesses the rest were mastitis cases [1]. Apart from the case which was reported in 2011 by Brncic et al.*,* in none of the reported non-typhoidal *Salmonella* breast abscess cases the patient had a history of gastrointestinal disease. In contrast, our patient had a history of gastroenteritis passed 2 months ago.

**Table 2:** Clinical features of reported cases of breast abscess due non-typhoidal *Salmonella* isolates. (Source: PubMed, Medline)

Year of publication	Reference number	Author	Patient age	Gender	Underlying condition	Species	Unilateral/bilateral breast abcess
1993	[1]	Edelstein et al.	28	Female	None	*Salmonella* serogroup B, serotype Reading	Unilateral
2000	[28]	Razeq et al.	47	Female	None	*Salmonella enterica* serotype Landwasser	Unilateral
2010	[8]	Benwan et al.	23	Female	None	*S. enterica* serotype Poona	Bilateral
2011	[7]	Brncic et al.	70	Male	Diabetes Mellitus Type 2	*S. enterica* serotype Enteritidis	Unilateral
2014	[29]	Mohamed and Asnis	66	Female	Breast cancer and bilateral breast implants	*S. enterica* serotype Enteritidis	Unilateral

## Conclusions

Although non-typhoidal *Salmonella* spp. are rare causes of breast abscess they should be kept in mind when a breast abscess occurs in a patient who have immune suppressive diseases or in a patient who use immunosuppressive drugs. Therefore, breast abscess material of these patients should be sent to the microbiology laboratory for proper diagnosis or diagnosis can be missed.

## Abbreviations

CCP, cyclic citrullinated peptide; CRP, C-reactive protein; EMB, eosin methylene blue; HBc, hepatitis B core; HBsAg, hepatitis B surface antigen; HCV, hepatitis C virus; HIV, human immunodeficiency virus; Maldi-TOF MS, matrix-assisted laser desorption/ionization mass spectrometry; RA, rheumatoid arthritis; RF, rheumatoid factor; USG, ultrasonography
